# A statistical method for predicting splice variants between two groups of samples using GeneChip^® ^expression array data

**DOI:** 10.1186/1742-4682-3-19

**Published:** 2006-04-07

**Authors:** Wenhong Fan, Najma Khalid, Andrew R Hallahan, James M Olson, Lue Ping Zhao

**Affiliations:** 1 Division of Public Health Sciences, Fred Hutchinson Cancer Research Center, 1100 Fairview Ave. N., Seattle, WA 98109, USA; 2 Clinical Research Division, Fred Hutchinson Cancer Research Center, 1100 Fairview Ave. N., Seattle, WA 98109, USA; 3 Department of Paediatrics and Child Health, University of Queensland, QLD, 4029, Australia

## Abstract

**Background:**

Alternative splicing of pre-messenger RNA results in RNA variants with combinations of selected exons. It is one of the essential biological functions and regulatory components in higher eukaryotic cells. Some of these variants are detectable with the Affymetrix GeneChip^® ^that uses multiple oligonucleotide probes (i.e. probe set), since the target sequences for the multiple probes are adjacent within each gene. Hybridization intensity from a probe correlates with abundance of the corresponding transcript. Although the multiple-probe feature in the current GeneChip^® ^was designed to assess expression values of individual genes, it also measures transcriptional abundance for a sub-region of a gene sequence. This additional capacity motivated us to develop a method to predict alternative splicing, taking advance of extensive repositories of GeneChip^® ^gene expression array data.

**Results:**

We developed a two-step approach to predict alternative splicing from GeneChip^® ^data. First, we clustered the probes from a probe set into pseudo-exons based on similarity of probe intensities and physical adjacency. A pseudo-exon is defined as a sequence in the gene within which multiple probes have comparable probe intensity values. Second, for each pseudo-exon, we assessed the statistical significance of the difference in probe intensity between two groups of samples. Differentially expressed pseudo-exons are predicted to be alternatively spliced. We applied our method to empirical data generated from GeneChip^® ^Hu6800 arrays, which include 7129 probe sets and twenty probes per probe set. The dataset consists of sixty-nine medulloblastoma (27 metastatic and 42 non-metastatic) samples and four cerebellum samples as normal controls. We predicted that 577 genes would be alternatively spliced when we compared normal cerebellum samples to medulloblastomas, and predicted that thirteen genes would be alternatively spliced when we compared metastatic medulloblastomas to non-metastatic ones. We checked the consistency of some of our findings with information in UCSC Human Genome Browser.

**Conclusion:**

The two-step approach described in this paper is capable of predicting some alternative splicing from multiple oligonucleotide-based gene expression array data with GeneChip^® ^technology. Our method employs the extensive repositories of gene expression array data available and generates alternative splicing hypotheses, which can be further validated by experimental studies.

## Background

Alternative splicing of pre-messenger RNA is an essential biological functional and regulatory component in higher eukaryotic cells. It increases the complexity of biological processes and gives the cells enhanced capability to respond to various factors, such as developmental changes and environmental stimuli. Some splice variants have been associated with diseases, such as mammary tumorigenesis [1] and ovarian cancer [2]. About 15% of single nucleotide mutations in the exon recognition process are associated with human genetic diseases [3]. Understanding the alternative splicing mechanism may also lead to finding potential treatments for related diseases [4].

In this paper, we describe a method for detecting alternative splicing variants using the GeneChip^® ^gene expression array data. Affymetrix GeneChip^® ^technology employs multiple probes per gene to measure gene expression. These multiple probes are short sequences located in different positions within each gene. Even though distributions of these probe sequences are not optimized for detecting alternative splicing, the probe sequence data obtained by the current GeneChip^® ^technology can be used to assess alternative splicing. In our method, we infer "pseudo-exons" from hybridization intensities of multiple probes that are spread over a probe set. A pseudo-exon is defined as a range of expressed sequence on the genome that we infer to be an exon based on probe intensities and physical adjacency.

**Figure 1:**
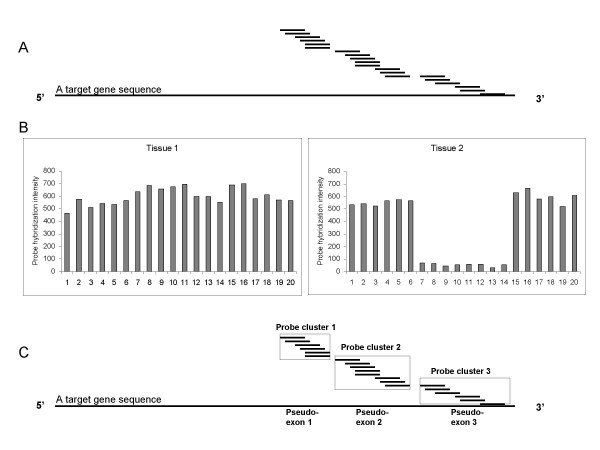
**A **Multiple probes are used to quantify the expression value for a gene in GeneChip^® ^technology. Currently the probe design has a 3' bias, i.e. probes are selected from the sequence at the 3'end of the gene. In the Hu6800 array, twenty probes are used for a single gene. 1 **B **Intensities of the twenty probes are plotted for both tissues 1 and 2. 1 **C **The twenty probes are clustered into three groups based on the similarity of probe intensity and probe adjacency. Each cluster, called a pseudo-exon in this paper, represents a sub-region of the gene.

Figure 1 illustrates how GeneChip^® ^expression array data can be used to detect alternative splicing. We show the probe locations for a hypothetical gene in Figure 1A and their corresponding hybridization intensities in Figure 1B. From the probe intensities, we infer that three clusters of probes represent three pseudo-exons (Figure 1C). For each of the pseudo-exons, we test whether the difference in probe intensities between tissue 1 and tissue 2 is significant. If the difference is statistically significant, we infer that there is alternative splicing between the two tissues for the region corresponding to the selected pseudo-exon. In our illustration, the region between probe #7 and probe #14, i.e. pseudo-exon 2 is predicted to be alternatively spliced between tissue 1 and tissue 2.

Previously, Hu et al reported a method, based on fold changes, to predict alternative splicing from GeneChip^® ^expression array data on ten tissue types [5]. For each probe, they calculated the difference in the fold change between each tissue type and the average of the remaining tissue types for the corresponding probe. If the fold change was greater than an empirically-determined threshold value R, they selected the gene sequence corresponding to that selected probe as an alternative splicing site for that tissue type. However, there are some problems with Hu's approach. First, the fold-change approach does not take into account sample variation and thus is less reliable when sample-to-sample variations are large. Second, their method is designed to predict splice variants in a dataset with multiple tissue types. Hu et al reported that prediction power decreased for a dataset that contained only three tissue types compared to a dataset that consisted of ten tissue types. The robustness of their method depended on the number of the tissue types in the dataset. Thus, their method is not suitable for the comparison of two tissue types such as detection of splice variants between two phenotypes, or two disease status, or two experimental stimuli.

In this paper, we propose an approach to predict splice variants between two groups of samples from GeneChip^® ^expression array data, taking into consideration sample variation. Our t-test based approach is more statistically vigorous and reliable than fold-change based methods. Furthermore, our method does not rely on a large number of tissue types. We implemented the method from Hu et al and compared the splice variants predicted from the two approaches. Our dataset consists of normal cerebellum, non-metastatic medulloblastomas, and metastatic medulloblastomas. The comparisons were made between normal cerebellum versus medulloblastomas, and non-metastatic medulloblastomas versus metastatic medulloblastomas.

## Results

### The computational algorithms

Our approach has two steps. In STEP 1, we infer pseudo-exons using multiple probe intensities. In STEP 2, we identify pseudo-exons that are differentially expressed between two groups of samples. In STEP 1, for each probe, we first compute the average of the difference in probe intensities between the two groups of samples. Then, based on the similarity of probe intensities and probe adjacency on the gene sequence, we merge probes into clusters that represent one pseudo-exon. In STEP 2, we test if the pseudo-exons are differentially expressed between the two groups of samples. The expression value from a pseudo-exon is treated as an entity in the current analysis, comparable to the gene expression from a complete probe set in customary analyses of gene expression data. The selected pseudo-exons are interpreted as an indication of alternative splicing at this region of the gene between the two comparison groups.

### Predicting splice variants between normal cerebellum and medulloblastomas

**Figure 2:**
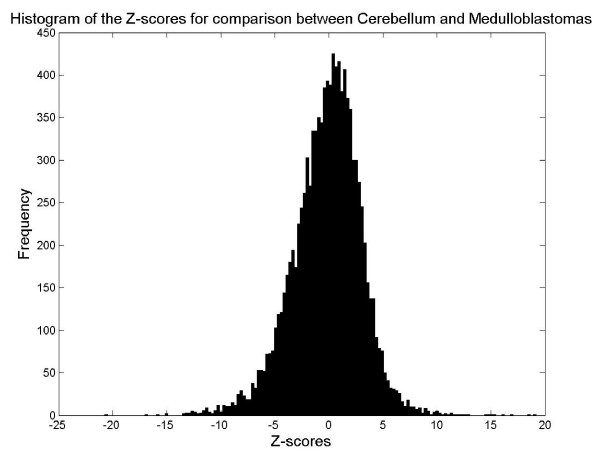
Histogram of the Z-scores for all 10,838 pseudo-exons obtained in the comparison of normal cerebellum samples with medulloblastomas.

For illustrative purposes, we applied the above method to predict splice variants between the normal cerebellum and medulloblastoma tumor samples, which included both non-metastatic and metastatic tumors. In STEP 1, using a significance level of 0.05 in the t-test, we identified 10,838 pseudo-exons out of a total of 142,580 (7129 × 20) probes that represent the 7,129 probe sets on the Hu6800 GeneChip^®^. In STEP 2, we compared the difference in expression values between the two groups for each pseudo-exon. The histogram of Z-scores from these tests is shown in Figure 2. With the significance threshold of the Z-score set to 4.8 (equivalent to one false positive error in the discovery), we discovered 811 pseudo-exons, derived from 577 genes, were significantly different between normal cerebellum and medulloblastoma tumor samples. Note that for some genes more than one pseudo-exon was selected.

### Predicting splice variants between non-metastatic medulloblastomas and metastatic medulloblastomas

Following the same procedure, we predicted splice variants between the non-metastatic and the metastatic medulloblastomas. We identified 8,319 pseudo-exons, thirteen of which were significantly different between non-metastatic and metastatic medulloblastomas (Table 1). Instead of conducting validation in a biological experiment, we searched two genome browsers for supportive evidence for our prediction. We input the thirteen genes in Table 1 into the Integrated Genome Browser (IGB) from Affymetrix [7] to see whether the probes in the identified pseudo-exons were positioned on separate exons within the same gene, which is a pre-requisite for alternative splicing. For further consistency, we checked whether the predicted pseudo-exons were reported as splice variants in the UCSC Human Genome Browser [8] under the track named "mRNA sequences from GenBank". In the IGB, we found four out of thirteen genes with predicted alternatively spliced pseudo-exons resided on different exons. These four genes were glutaredoxin (GLRX), carboxypeptidase N polypeptide 1 (CPN1), Keratin 7 (KRT7) and killer cell lectin-like receptor subfamily C member 3 (KLRC3). For instance, we predicted the last three probes for GLRX were within one pseudo-exon. In IGB, based on RefSeq information, these three probes are on a different exon. We searched alternatively transcribed variants deposited in GenBank in the "mRNA sequences from GenBank" track in UCSC Human Genome Browser for the genes confirmed by IGB. All of them except for CPN1 have at least two transcript sequences in the GenBank database. At least one of these sequences lack the region that we predicted to be alternatively spliced, and at least one of these sequences contain the predicted region. We also searched PubMed for reported splice variants for the thirteen identified genes. Five of out of the thirteen genes were reported in the literature to have splice variants. They are nitric oxide synthase 1 (NOS1) [9], low density lipoprotein receptor (LDLR) [10], thrombopoietin (THPO) [11], Down syndrome critical region gene 1 (DSCR1) [12], paired box gene 2 (PAX2) [13].

**Table 1:** Alternative spliced genes selected by our method: Comparison of non-metastatic medulloblastomas with metastatic medulloblastomas

Affymetrix Probe Set ID	Gene Symbol	Number of Affymetrix Probes in the Predicted Pseudo-exon	Nucleotide Positions of Predicted Pseudo-exon in the Gene	Mean Difference	Standard Error	Z-score	Description of the Genes
M81882_at	GAD2	4	(2135–2285)	-1.28	0.20	-6.45	glutamate decarboxylase 2 (pancreatic islets and brain, 65 kDa)
M13955_at	KRT7	5	(1402–1474)	-0.63	0.12	-5.23	keratin 7
U17327_at	NOS1	7	(6805–7003)	-0.66	0.13	-5.19	nitric oxide synthase 1 (neuronal)
X14329_at	CPN1	4	(1569–1665)	-0.62	0.12	-5.18	carboxypeptidase N, polypeptide 1, 50 kD
M89470_s_at	PAX2	6	(2855–2972)	-0.92	0.19	-4.91	paired box gene 2
L14542_at	KLRC3	5	(916–1006)	-1.18	0.24	-4.91	killer cell lectin-like receptor subfamily C, member 3
X76648_at	GLRX	3	(704–776)	-1.35	0.28	-4.86	glutaredoxin (thioltransferase)

U82987_at	BBC3	3	(1578–1638)	2.25	0.32	6.98	BCL2 binding component 3
U01102_at	SCGB1A1	2	(409–439)	1.42	0.25	5.62	secretoglobin, family 1A, member 1 (uteroglobin)
M28219_at	LDLR	15	(67–277)	0.77	0.14	5.42	low density lipoprotein receptor (familial hypercholesterolemia)
X68194_at	SYPL	5	(1915–2089)	1.67	0.31	5.42	synaptophysin-like protein
U85267_at	DSCR1	10	(64–169)	1.20	0.24	5.08	Down syndrome critical region gene 1
L36051_at	THPO	6	(1647–1809)	1.05	0.21	4.96	thrombopoietin (myeloproliferative leukemia virus oncogene ligand, megakaryocyte growth and development factor)

Number of Affymetrix Probes in the Predicted Pseudo-exon: number of probes that are contained in a predicted alternatively spliced pseudo-exon. Nucleotide Positions of Predicted Pseudo-exon in the Gene: nucleotide positions of the pseudo-exon from the beginning of the gene it resides. Mean difference: Mean difference of the expression values between the two tissue types being compared for each predicted pseudo-exon in the t-test in STEP 2. Standard Error: the standard error calculated in the same t-test. Z-score: the ratio of mean difference over standard error (noise), a measure of significance of the difference between the two tissues being compared. The sign of the Z-scores indicate direction of the difference. A negative Z-score means a lower expression in metastatic medulloblastomas than in non-metastatic medulloblastomas, and vice-versa for a positive Z-score.

### Comparison with Hu et al's approach

To compare our method with the Hu et al's, we implemented their method and applied it to our dataset. When comparing normal cerebellum and medulloblastomas samples using Hu et al's method, we inferred 31 alternatively spliced genes with the selection criterion used by Hu et al in their paper (Table 2). Among these 31 genes, seven overlapped with the findings from our approach (Table 3). For four of them, D87119_at, U14971_at, U29953_rna1_at, X04828_at, the locations of the alternative splicing were consistent between the two methods. In the comparison between non-metastatic and metastatic medulloblastoma samples, we did not find any gene that was alternatively spliced by Hu et al's method. We also investigated the effect of different selection criteria in Hu et al's method (i.e. the R threshold, which is the ratio of the probe intensity in a tissue over the mean of the probe intensities in the remaining nine tissue types for the same probe). Table 4 shows the relation between the 577 genes predicted by our approach and the genes selected with different R thresholds in Hu's approach. Numbers of predicted alternatively spliced genes increase as smaller R values (less stringent) are used.

**Table 2:** Alternative spliced genes inferred by applying Hu's method to our dataset: Comparison of normal cerebellum with medulloblastoma samples

Affy Probe Set ID	Gene Symbol	Number of Affymetrix Probes in the Predicted Pseudo-exon	Nucleotide Positions of Predicted Pseudo-exon in the Gene	Description of the Genes
X51362_s_at	DRD2	2	(2541–2574)	dopamine receptor D2
M15517_cds5_at	TTR	3	(155–197)	transthyretin (prealbumin, amyloidosis type I)
Y10141_s_at	SLC6A3	2	(96–125)	solute carrier family 6 (neurotransmitter transporter, dopamine), member 3
Z14982_rna1_at	PSMB8	2	(820–850)	proteasome (prosome, macropain) subunit, beta type, 8 (large multifunctional protease 7)
X69654_at	RPS26	2	(9–35)	ribosomal protein S26
U63842_at	NEUROG1	2	(834–891)	neurogenin 1
M97815_at	CRABP2	2	(524–554)	cellular retinoic acid binding protein 2
D00017_at	ANXA2	2	(1229–1265)	annexin A2
U13021_s_at	CASP2	3	(844–913)	caspase 2, apoptosis-related cysteine protease (neural precursor cell expressed, developmentally down-regulated 2)
U30999_at	ALCAM	2	(373–403)	activated leukocyte cell adhesion molecule
X04828_at	GNAI2	3	(1668–1701)	guanine nucleotide binding protein (G protein), alpha inhibiting activity polypeptide 2
U14971_at	RPS9	2	(319–373)	ribosomal protein S9
U79299_at	OLFM1	2	(1342–1372)	olfactomedin 1
L20298_at	CBFB	2	(2298–2334)	core-binding factor, beta subunit
X93017_at	SLC8A3	2	(1725–1821)	solute carrier family 8 (sodium-calcium exchanger), member 3
M17886_at	RPLP1	2	(127–163)	ribosomal protein, large, P1
D16480_at	HADHA	2	(2335–2365)	hydroxyacyl-Coenzyme A dehydrogenase/3-ketoacyl-Coenzyme A thiolase/enoyl-Coenzyme A hydratase (trifunctional protein), alpha subunit
D38305_at	TOB1	2	(707–749)	transducer of ERBB2, 1
U32519_at	G3BP	2	(1534–1564)	Ras-GTPase-activating protein SH3-domain-binding protein
U07919_at	ALDH1A3	3	(3363–3411)	aldehyde dehydrogenase 1 family, member A3
U29953_rna1_at	SERPINF1	2	(1288–1324)	serine (or cysteine) proteinase inhibitor, clade F (alpha-2 antiplasmin, pigment epithelium derived factor), member 1
D55716_at	MCM7	2	(2288–2396)	MCM7 minichromosome maintenance deficient 7 (S. cerevisiae)
J05448_at	POLR2C	2	(1575–1605)	polymerase (RNA) II (DNA directed) polypeptide C, 33 kDa
U46570_at	TTC1	2	(1226–1262)	tetratricopeptide repeat domain 1
D87119_at	TRB2	2	(4022–4136)	tribbles homolog 2
X69910_at	CKAP4	2	(2543–2573)	cytoskeleton-associated protein 4
U50078_at	HERC1	2	(14885–14915)	hect (homologous to the E6-AP (UBE3A) carboxyl terminus) domain and RCC1 (CHC1)-like domain (RLD) 1
J04164_at	IFITM1	2	(798–828)	interferon induced transmembrane protein 1 (9–27)
AFFX-HUMRGE/M10098_3_at	N/A	2	(1562–1613)	N/A
HG2788-HT2896_at	N/A	2	(N/A-N/A)	N/A
HG2994-HT4850_s_at	N/A	2	(N/A-N/A)	N/A

Number of Affymetrix Probes in the Predicted Pseudo-exon: number of probes that are contained in a predicted alternatively spliced pseudo-exon. Nucleotide Positions of Predicted Pseudo-exon in the Gene: nucleotide positions of the pseudo-exon from the beginning of the gene it resides. Mean difference: Mean difference of the expression values between the two tissue types being compared for each predicted pseudo-exon in the t-test in STEP 2. Standard Error: the standard error calculated in the same t-test. Z-score: the ratio of mean difference over standard error (noise), a measure of significance of the difference between the two tissues being compared. The sign of the Z-scores indicate direction of the difference. A negative Z-score means a lower expression in metastatic medulloblastomas than in non-metastatic medulloblastomas, and vice-versa for a positive Z-score.

**Table 3:** Overlapping of the predicted gene from our method and Hu's method for the comparison of normal cerebellum and medulloblastoma samples

Affy Probe Set ID	Gene Symbol	Number of Affymetrix Probes in the Predicted Pseudo-exon		Nucleotide Positions of Predicted Pseudo-exon in the Gene		Descriptions of the Genes
		Ours	Hu's	Ours	Hu's	
X04828_at*	GNAI2	3	3	(1668–1701)	(1668–1701)	guanine nucleotide binding protein (G protein), alpha inhibiting activity polypeptide 2
U14971_at*	RPS9	19	2	(103–685)	(319–373)	ribosomal protein S9
U29953_rna1_at*	SERPINF1	13	2	(1288–1492)	(1288–1324)	serine (or cysteine) proteinase inhibitor, clade F (alpha-2 antiplasmin, pigment epithelium derived factor), member 1
D87119_at*	TRB2	13	2	(3824–4184)	(4022–4136)	tribbles homolog 2
X69910_at	CKAP4	5	2	(2789–2891)	(2543–2573)	cytoskeleton-associated protein 4
U30999_at	ALCAM	16	2	(25–337)	(373–403)	activated leukocyte cell adhesion molecule
D55716_at	MCM7	8	2	(1952–2096)	(2288–2396)	MCM7 minichromosome maintenance deficient 7 (S. cerevisiae)

* Consistent alternative splice sites between two methods.

**Table 4:** Comparison of the results from our approach and those from Hu's using different R thresholds when normal cerebellum samples are compared with medulloblastomas

R used	Number of Genes Found in Hu's Approach	Number of Overlap Between Hu's and Our 577 Genes	Percentage of the overlapping genes based on number of genes found in Hu's method	Percentage of the overlapping genes based on our 577 selected genes
4	324	69	21%	11.9%
6	103	28	27%	4.9%
8	53	14	26%	2.4%
10	31	7	23%	1.2%

Genes found in Hu's methods using different R thresholds are compared to each other. Larger R value represents more stringent selection criterion. Genes found using smaller R values always include those found using larger R values, i.e. gene list of 324 genes contains gene list of 103 genes, etc. Genes obtained from Hu's method are also compared with 577 genes from our approach. Numbers of overlapping genes are presented in the third column for different R values. Similarly, overlapping genes for the smaller R values contains those for the larger R values, i.e. gene list of 69 genes contains gene list of 28 genes, etc.

We checked both IGB and UCSC Human Genome Browsers for supportive evidence for the seven predicted alternatively spliced variants in Table 3. We found four genes that had predicted pseudo-exons located on separate exons according to IGB and alternative spliced mRNA from GenBank in UCSC Human Genome Browser. They are guanine nucleotide binding protein alpha inhibiting activity polypeptide 2 (GNAI2), ribosomal protein S9 (RPS9), activated leukocyte cell adhesion molecule (ALCAM), and minichromosome maintenance deficient 7 (MCM7). There are splicing variants reported in PubMed literature for ALCAM [14].

## Discussion

We have developed a two-step approach to predict splice variants between two groups of samples using GeneChip^® ^gene expression array data. We illustrated the method using empirical data from normal cerebellum, metastatic medulloblastoma and non-metastatic medulloblastoma samples. We predicted a total of 577 alternatively spliced genes when we compared normal cerebellum with medulloblastomas tumor samples and thirteen alternatively spliced genes when we compared non-metastatic medulloblastomas with metastatic medulloblastomas. A comparison of the results from our approach and the method described by Hu et al on the same dataset revealed some overlapping alternatively spliced genes.

Our proposed method can be used to predict splice variants and takes advantage of the extensive repositories of gene expression array data. Inferred splice variants can be used to generate alternative splicing hypotheses for subsequent experimental validation. Higher signal quality in the newer generation GeneChip^®^, such as U133 Plus 2.0 array, should make our predictions more robust. Recently, a genome-wide human exon array became available from Affymetrix [7] to detect known alternative splicing in a biological sample. Bypassing the need for defining "pseudo-exons" in the STEP 1 of our approach, one can directly use STEP 2 of our method to predict splice variants. As expected, such an exon array coupled with our rigorous statistical method may improve the power to predict more splice variants.

There are some limitations associated with using GeneChip^® ^gene expression array data to detect alternatively spliced variants. Currently, GeneChip^® ^probes cover 600 base pairs in sequence from the 3' end. Thus we can only detect splice variants at the 3' end. Furthermore, some 3' end splice variants could be due to alternative polyadenylation sites, and our method does not differentiate between these in the analysis. The splice variants resulting from the 3' non-translational region could be removed by checking whether the predicted pseudo-exons on the 3' end are located in translational regions.

Since our approach depends on probe intensities to cluster probes into pseudo-exons within a single gene, non-specific hybridization in an expression array could complicate this step (STEP 1), thus result in both false positive and false negative findings. Cross-hybridization can be partially addressed by excluding lower grade probe sets, such as probe sets with the suffix _s or _x, which could hybridize to multiple genes either before analysis or from the gene list after analysis.

## Conclusion

In this paper we describe a method that can generate hypotheses of alternative splicing for further investigation. Our approach overcomes two limitations of a previously proposed method [5]: 1) we use t-tests instead of fold changes, 2) we can predict splicing variants between two groups of samples. These differences make our inference more robust and not dependent on multiple tissue types to stabilize the inference.

## Methods

### Dataset

Our empirical dataset consists of GeneChip^® ^Hu6800 expression array data from sixty-nine medulloblastoma samples and four cerebellum samples as normal controls. Among the medulloblastoma samples, forty-two are from non-metastatic tumors and twenty-seven are from metastatic tumors. There are 7,129 probe sets in the Hu6800 expression array, and twenty probes in each probe set.

### Inferring pseudo-exons within a gene (STEP 1)

In this step, we merge probes within a gene into clusters that represent pseudo-exons. First, we compute the difference in probe hybridization intensity between two groups of samples for each probe. Then, for each gene, we merge probes into clusters based on the similarity of the differences in probe intensity (between the two groups of samples) and the probe adjacency on the genome sequence. For a gene, let ***Y***_(*i*, 1)_,  and *n*_1 _be the probe intensity for the *i*th probe in sample group 1, variance, and sample size, respectively. Similarly, ***Y***_(*i*, 2)_,  and *n*_2 _are for the sample group 2. Within the gene, the index *i *increases from the direction of the 5' end to the 3' end. We start with the first probe from the 5' end and compute:





where  is the mean of probe intensities. If the absolute value of ***t***_i _does not exceed the threshold value at the significance level *α *= 0.05, we merge the *i*th probe with the (*i*+1)th probe to generate a pseudo-exon. The resulting pseudo-exon becomes the new *i*th probe in the next iteration of the t-test. The pseudo-exon extends with each iteration until the results of the t-test become significant or reach the last probe within a probe set. If ***t***_*i *_exceeds the significance threshold value, we do not merge the *i*th probe with the (*i*+1)th probe, but start generating a new pseudo-exon from this (*i*+1)th probe, using the same iteration procedure. After we finish the last probe at the 3' end, we may either have several pseudo-exons or only one pseudo-exon (i.e. the entire probe set) if every t-statistic within a probe set is not significant.

### Testing for statistical significance (STEP 2)

For each pseudo-exon, we determine whether there is a difference in hybridization intensity between the two groups x_1 _and x_2_. Our null hypothesis is that, for any pseudo-exon, the difference in probe intensity between the two groups is zero. If we reject the null hypothesis for a pseudo-exon, meaning that the hybridization intensities between the two groups are significant different for that pseudo-exon, we then infer that there is a splice variant between the two groups of samples for the corresponding region within the gene.

In the same vein as Li and Wong's model to analyze gene expression at the probe level [15], we propose a multiplicative heterogeneity factor model to associate the probe intensities of a pseudo-exon directly with the covariate, i.e. group indictor x_*k*_:



where *Y*_*jik *_is the hybridization intensity for the *i*th probe in the *j*th pseudo-exon in the *k*th sample, N is the number of probes in the *j*th pseudo-exon, *δ*_*k *_and *λ*_*k *_are heterogeneity factors for normalization,*x*_*k *_is the group indicator for the *k*th sample, *β*_*j *_is the coefficient for *j*th pseudo-exon, *φ*_*ji *_is the multiplicative probe-specific parameter for *i*th probe in *j*th pseudo-exon, and *ξ *is random variation term. To avoid making any distributional assumptions, we applied estimating equation techniques to estimate the coefficients and their standard errors for making statistical inferences [16-19].

## Abbreviations

IGB: Integrated Genome Browser; UCSC: University of California, Santa Cruz

## Competing interests

The author(s) declare that they have no competing interests.

## Authors' contributions

WF performed the data analysis, drafted the manuscript and developed method jointly with LPZ. NK revised the manuscript. ARH and JMO conceived the study. LPZ conceived the study and developed the method jointly with WF. All authors read and approved the final manuscript.

## Supplementary Material

Additional File 1 Alternative spliced pseudo-exons selected by our method: Comparison of normal cerebellum with medulloblastomas. Complete results for the 811 pseudo-exons predicted to be alternatively spliced between normal cerebellum and medulloblastomas. Click here for file
